# Unveiling the MIL-53(Al) MOF: Tuning Photoluminescence and Structural Properties via Volatile Organic Compounds Interactions

**DOI:** 10.3390/nano14050388

**Published:** 2024-02-20

**Authors:** Tanzeel Ul Rehman, Simonpietro Agnello, Franco Mario Gelardi, Martina Maria Calvino, Giuseppe Lazzara, Gianpiero Buscarino, Marco Cannas

**Affiliations:** Dipartimento di Fisica e Chimica−Emilio Segrè, Università degli Studi di Palermo, 90123 Palermo, Italy; tanzeelul.rehman@unipa.it (T.U.R.); simonpietro.agnello@unipa.it (S.A.); franco.gelardi@unipa.it (F.M.G.); martinamaria.calvino@unipa.it (M.M.C.); giuseppe.lazzara@unipa.it (G.L.); gianpiero.buscarino@unipa.it (G.B.)

**Keywords:** Metal-Organic Frameworks, MIL-53(Al), guest-adsorption, structural transitions, photoluminescence, VOCs

## Abstract

MIL-53(Al) is a metal-organic framework (MOF) with unique properties, including structural flexibility, thermal stability, and luminescence. Its ability to adsorb volatile organic compounds (VOCs) and water vapor makes it a promising platform for sensing applications. This study investigated the adsorption mechanism of MIL-53(Al) with different VOCs, including ketones, alcohols, aromatics, and water molecules, focusing on structural transformations due to pore size variation and photoluminescence properties. The reported results assess MIL-53(Al) selectivity towards different VOCs and provide insights into their fundamental properties and potential applications in sensing.

## 1. Introduction

The field of porous materials has experienced significant progress in recent decades, with Metal-Organic Frameworks (MOFs) emerging as a leading class of materials in scientific exploration, blending diverse disciplines such as materials science, chemistry, and physics [1,2,3]. MOFs are crystalline materials constructed from metal ions or clusters bridged by organic linkers, which create a network of pores with exceptionally high surface areas [4,5,6]. A fascinating subclass of MOFs exhibits luminescence properties that, in combination with the inherent structural flexibility, are useful in a wide range of potential applications in optoelectronic devices, lighting, and biological imaging [7,8,9,10,11,12]. In fact, the adaptability of the MOF structure as a response to changes in their environment confers ratiometric fluorescence properties, which can be very advantageous for the detection of specific analytes with high sensitivity [13]. In this emerging field, the intricate interplay between luminescence and flexibility within MOFs holds great promise for transformative advancements in areas such as environmental monitoring, healthcare diagnostics, and beyond [14,15,16,17].

Among the various luminescent MOFs, MIL-53(Al) has attracted significant attention due to its ability to undergo structural transformations between large pore (LP) and narrow pore (NP) in response to external stimuli, such as changes in temperature, pressure, and guest molecule adsorption [18,19,20]. This distinguishing characteristic to expand or contract its framework to accommodate guest molecules while maintaining its structural integrity has sparked significant interest within the scientific community and prompted further exploration of the underlying mechanisms that govern such transformations [21,22]. The main focus of this work is the MIL-53(Al)’s response to volatile organic compounds (VOCs), which are prevalent indoor air pollutants that contribute to air quality degradation and are found in sources such as industrial emissions, vehicle exhaust and household products [23,24]. The adsorption and removal of these compounds are crucial for preventing negative health effects and environmental impact [25]. Therefore, a thorough investigation of MIL-53(Al)’s structural transformations when faced with different VOCs is necessary to fully utilize its potential in VOC capture and remediation [26,27,28,29,30]. It is worth noting that as a luminescent sensor for VOCs, MIL-53(Al) has several benefits over other materials characterized by some drawbacks, such as limited sensitivity, selectivity, and adaptability. In addition to the structural flexibility that improves its ability to effectively accommodate different VOCs, MIL-53(Al) demonstrates luminescent characteristics that are modulated by the presence of guest molecules, rendering it appropriate for use in sensing applications with high selectivity [30]. Moreover, its huge surface area and porous structure also offer plenty of room for VOC adsorption, which improves the sensor’s sensitivity.

In this work, we will conduct a thorough investigation into the adsorption response of MIL-53(Al), focusing on several critical aspects. First, we will carefully examine the structural transitions that occur when different VOCs and water molecules are introduced to MIL-53(Al), utilizing complementary characterization techniques to unveil the intricacies of these transformations. Second, we will delve into the photoluminescence properties of MIL-53(Al) and analyze how its luminescence responds to the presence of guest molecules, shedding light on its potential as a ratiometric fluorescence sensor. Additionally, we will explore the realm of sorption kinetics and attempt to unravel the dynamic adsorption process within MIL-53(Al), underscoring its significance for real-time sensing applications. Lastly, we will evaluate the selective sensing capabilities of MIL-53(Al) regarding various VOCs and water vapor, ultimately providing valuable insights into its capacity for distinguishing between different analytes. This comprehensive analysis aims to enhance our understanding of MIL-53(Al)’s interactions with guest molecules and its promising prospects in the domain of selective and responsive sensing technologies.

## 2. Materials and Methods

MIL-53(Al) and its organic linker, named terephthalic acid (BDC, 99%+), were purchased from Sigma-Aldrich. (Sigma-Aldrich S.r.l., Milan, Italy) Moreover, volatile organic compounds, such as toluene (C7H8, 99%), ethanol (C2H5OH, 99%), methanol (CH3OH, 99%), isopropanol (C3H7OH, 99%), and acetone (CH3COCH3, 99%) were provided from Fisher Scientific (Thermo Fisher Scientific S.p.a., Milan, Italy). Two distinct series of samples were prepared: the first involved activating the raw powder at 300 °C in a glass tube for 70 h, followed by sealing it (denoted as MIL-53(Al)/activated); the second was prepared by adsorbing water molecules and different volatile organic compounds (VOCs) onto the activated MIL-53(Al) (denoted as MIL-53(Al)/hydrated and MIL-53(Al)/VOCs). For the experiments carried out in this work, six samples of MIL-53(Al) were prepared using an equal amount (100 mg) of MOF powder and soaked in an equal quantity (2 mL) of water and five different VOCs solvents (Toluene, Acetone, Ethanol, Methanol and Isopropanol) and dried at ambient conditions. Moreover, one sample of terephthalic acid was also prepared as a comparison. As will be reported in the results session; we did not perform any guest adsorption experiments on BDC linkers that were investigated just for comparison with the pristine MOF sample.

The crystalline structure of MIL-53(Al) was characterized using powder X-ray diffraction (PXRD) using a Rigaku Miniflex diffractometer (Rigaku, assing S.p.a., Roma, Italy) with a Cu Kα source (1.541 Å). We collected diffraction data at a 0.01° step size, 1°/min, with a range of 2θ angles from 7° to 70°. To investigate the thermal stability of MIL-53(Al), we performed thermogravimetric analysis (TGA). The experiments were conducted on a TGA 550 (Discovery Series–TA Instruments) (TAinstruments, Milan, Italy), with each sample heated in a platinum pan from room temperature to 800 °C at a scanning rate of 20 °C/min.

The effect of guest molecules on the vibrational spectra of MIL-53(Al) was studied using FT-Raman spectroscopy (Bruker Scientific LLC, Billerica, MA, USA). Our experiments were carried out using a Bruker Vertex 70v RAMII spectrometer (Bruker Scientific LLC, Billerica, MA, USA) using a Nd:YAG laser with a wavelength of 1064 nm and a power of 500 mW as the excitation source. The spectra reported in this work were recorded with a spectral resolution of 3 cm^−1^, averaging over 200 scans.

Time-resolved photoluminescence spectroscopy (TRPL) (Teledyne Princeton Ins., Trenton, NJ, USA) was used to investigate the fluorescent properties and lifetimes of solid-state samples of MIL-53(Al). The excitation source consisted of an optical parametric oscillator (VIBRANT OPOTEK) pumped by the third harmonic (3.49 eV) of a Nd:YAG laser with a pulse width of 5 ns and a repetition rate of 10 Hz. The emitted light was analyzed using a monochromator equipped with a grating of 150 lines/mm and blaze wavelength of 300 nm and acquired using an intensified CCD camera (Teledyne Princeton Ins., Trenton, NJ, USA) that was driven by a delay generator (PIMAX Princeton Instruments) (Teledyne Princeton Ins., Trenton, NJ, USA). We set the acquisition time window (TW) and delay (TD) with respect to the laser pulses and detected the emission spectra with a bandwidth of 5 nm while correcting for the monochromator dispersion. 

## 3. Results and Discussion

### 3.1. Structural Properties of MIL-53(Al) MOFs

**Figure 1:**
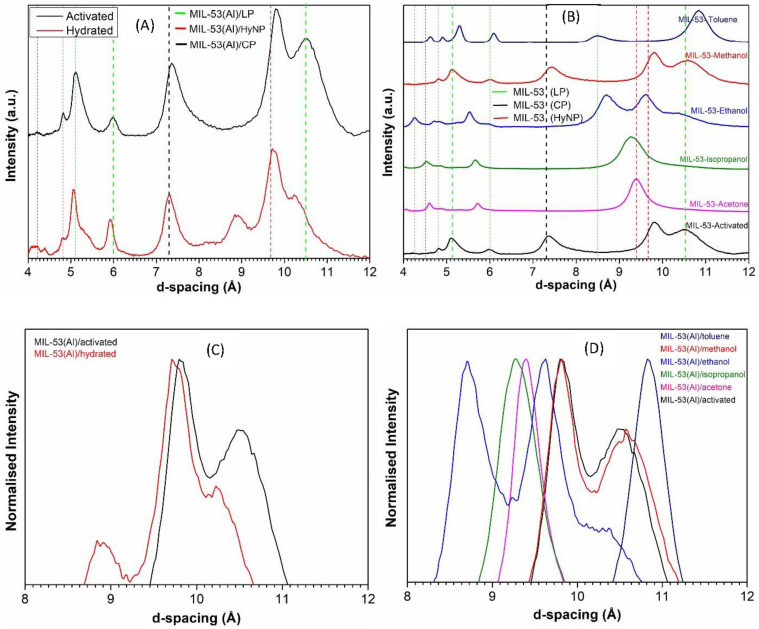
PXRD patterns of MIL-53(Al)/activated and MIL-53(Al)/hydrated (**A**); PXRD patterns of MIL-53(Al)/VOCs with the dashed lines representing the positions of the peaks associated with the three possible structures of MIL-53(Al) (**B**). Zoomed view of PXRD patterns of MIL-53(Al)/activated and MIL-53(Al)/hydrated (**C**). Zoomed view of PXRD patterns of MIL-53(Al)/VOCs comparison with MIL-53(Al)/activated (**D**).

Structural properties of MIL-53(Al)/activated, MIL-53(Al)/hydrated, and MIL-53(Al)/VOCs are evidenced by PXRD spectra in Figure 1A. To enhance the scope of our analysis, we have also compared these findings with relevant data from the scientific literature, which are represented as vertical dashed lines in the same Figure [31,32,33,34]. Henceforth, the PXRD characteristics related to the three possible structures of MIL-53(Al) are the following: for MIL-53(Al)/LP, the main peaks originate at the d-spacing of 10.50 Å, 8.46 Å, 6.0 Å, and 5.12 Å, as represented with green color vertical dashed lines; for MIL-53(Al)/hyNP the peaks are at 9.66 Å, 9.37 Å, 4.94 Å, and 4.25 Å, as displayed by red color dashed lines; for MIL-53(Al)/CP the peaks are at 7.30 Å, and 4.70 Å, as identified with black color vertical dotted line. This analysis indicates that MIL-53(Al)/activated mainly exhibits the large pore (LP) structure, with minor traces of closed pore (CP) and hydrated narrow pore (HyNP) structures. Conversely, the MIL-53(Al)/hydrated sample displays a unique crystalline composition, with large pore (LP) and hydrated narrow pore (HyNP) structures present in roughly equal proportions. Indeed, the contribution of the closed pore (CP) structure is marginal in this sample.

The identification of these structures (LP, CP, HyNP) via PXRD is important to determine the response of MIL-53(Al) to different VOCs, as shown in Figure 1B. When exposed to acetone and isopropanol, MIL-53(Al) mainly retains the hydrated narrow pore (HyNP) structure. In the presence of ethanol, a considerable contribution from the hydrated narrow pore (HyNP) structure is observed alongside the fractional contribution of the large pore (LP) and closed pore (CP) structures. When exposed to methanol, MIL-53(Al) notably exists in large pore (LP) structures with a minimal presence of both hydrated narrow pore (HyNP) and closed pore (CP) structures. Lastly, in the presence of toluene, the majority of the phase maintains the large pore (LP) form, with a phase transition occurring at higher d-spacing. For the sake of clarity, in Figure 1C,D, we have also presented the zoomed-in normalized diffraction patterns (from 8 to 12 Å) of MIL-53(Al)/activated, MIL-53(Al)/hydrated, and MIL-53(Al)/VOCs, respectively. Moreover, for our convenience, we also calculated the crystallinity index of all these samples by using the following relation:

$$\mathrm{Crystallinity}\;\mathrm{Index}\;(\mathrm{CI})=\frac{\mathrm{Total}\;\mathrm{area}\;\mathrm{of}\;\mathrm{the}\;\mathrm{crystalline}\;\mathrm{peaks}}{\mathrm{Total}\;\mathrm{area}\;\mathrm{of}\;\mathrm{the}\;\mathrm{crystalline}+\mathrm{amorphous}\;\mathrm{peaks}}$$

MIL-53(Al)/activated, and MIL-53(Al)/hydrated possessed a CI = 90 ± 1% and CI = 78 ± 1%, respectively. In the case of VOCs, MIL-53(Al)/isopropanol and MIL-53(Al)/acetone notably displayed a smaller CI, the order of 74 ± 1% and 76 ± 1%, while MIL-53(Al)/methanol presented a CI = 85 ± 1%. On the other hand, MIL-53(Al)/ethanol and MIL-53(Al)/toluene showcased a higher CI of 88 ± 1% and 90 ± 1%, respectively. These findings indicate the varying degrees of crystallinity in MIL-53(Al) samples under distinct adsorption compounds, providing considerable insights into the structural transformations brought about by the presence of different VOCs. 

### 3.2. Thermal Properties of MIL-53(Al) MOFs

**Figure 2:**
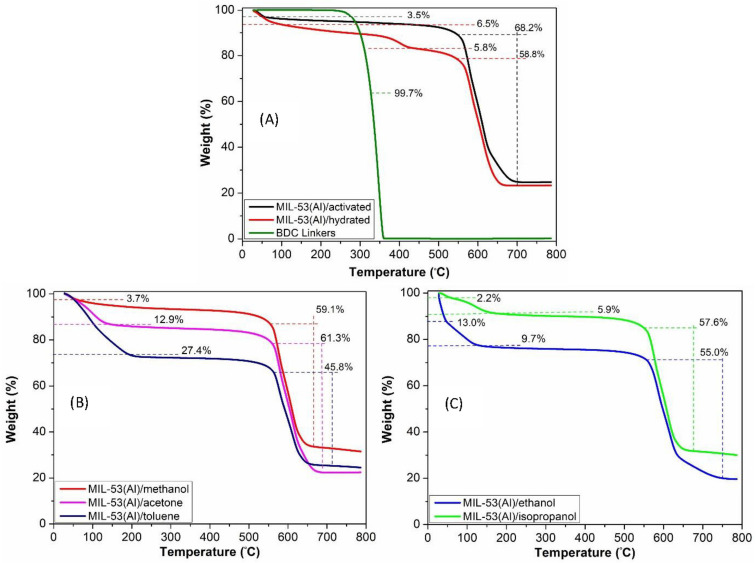
TGA analysis of free BDC linkers and MIL-53(Al)/activated and hydrated (**A**), MIL-53(Al)/methanol/acetone and toluene (**B**) and MIL-53(Al)/ethanol and isopropanol (**C**).

Figure 2 shows the TGA analysis of MIL-53(Al) MOF and its responses when adsorbed with five different VOCs. As shown in Figure 2A, the activated MIL-53(Al) MOF exhibits characteristic weight loss patterns, with an initial loss attributed to moisture and impurity desorption below 100 °C, followed by significant degradation of the MOF framework above 550 °C [35,36]. Instead, MIL-53(Al)/hydrated shows a three-step weight loss, with further weight loss below 350 °C, corresponding to the decomposition of organic linkers. To support this hypothesis, TGA analysis of free BDC linkers is also reported and points out that they are totally decomposed above 300 °C. These variations can be attributed to the interactions between the adsorbates and the MOF, which influence the MOF’s thermal stability and degradation behavior. Figure 2B,C evidence that when adsorbed with various VOCs, MIL-53(Al) exhibited distinct weight loss profiles. MIL-53(Al)/toluene led to a two-step weight loss, suggesting the desorption of toluene molecules below 200 °C and framework degradation above 550 °C. Similar patterns were observed for MIL-53(Al)/methanol and acetone adsorption as well. However, isopropanol and ethanol adsorption resulted in an additional weight loss step below 200 °C, indicating the desorption of strongly interacted guest molecules within the MOF cavities. Furthermore, from the initial weight losses (below 200 °C), we have also estimated the loading quantity of VOCs inside the MOF with an uncertainty of 10%: according to our findings, there were 2.0 µmol of methanol, 18 µmol of ethanol, 3.0 µmol of isopropanol, 4.0 µmol of acetone, and 6.0 µmol of toluene, respectively.

### 3.3. FT-Raman Spectroscopy of MIL-(Al) MOFs

**Figure 3:**
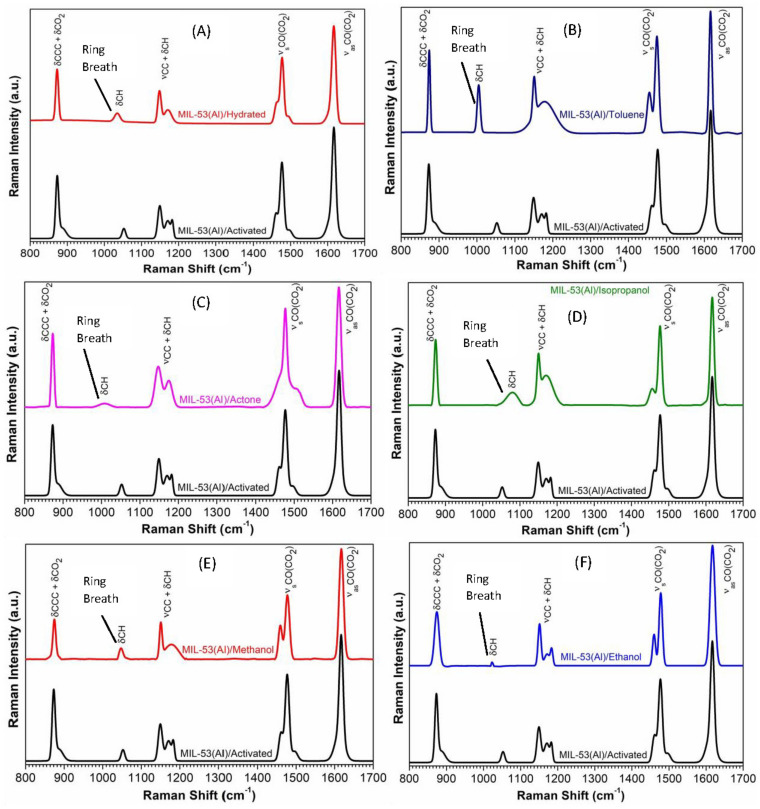
Comparison of FT-Raman spectrum acquired in MIL-53(Al)/activated with MIL-53(Al)/hydrated (**A**), MIL-53(Al)/toluene (**B**), MIL-53(Al)/acetone (**C**) MIL-53(Al)/isopropanol (**D**), MIL-53(Al)/methanol (**E**) and MIL-53(Al)/ethanol (**F**).

The FT-Raman spectra of MIL-53(Al) in various states, including activated, hydrated, and with VOCs, are presented in Figure 3. For the sake of comparison, all figures are shown with the spectrum of MIL-53(Al)/activated. The most prominent features associated with the carboxylate groups are the highly asymmetric ν_as_CO(CO_2_))-band, which is observed at a wavenumber of 1617 cm^−1^ and dominates the entire spectrum, and the symmetric vibrations at 1477 cm^−1^. Another significant spectral feature is the band at 1149 cm^−1^, which is attributed to the stretching mode associated with the carboxyl group and the rocking of hydrogen atoms on the aromatic ring (νCC + δCH). Additionally, the appearance of a band at 873 cm^−1^ is linked to the bending of the aromatic ring and the carboxyl group of the organic linker (δCCC + δCO_2_) [37]. It is worth noting that the intensities and shapes of these Raman peaks are influenced by the specific guest molecules and the dynamic host-guest interactions they participate in.

**Figure 4:**
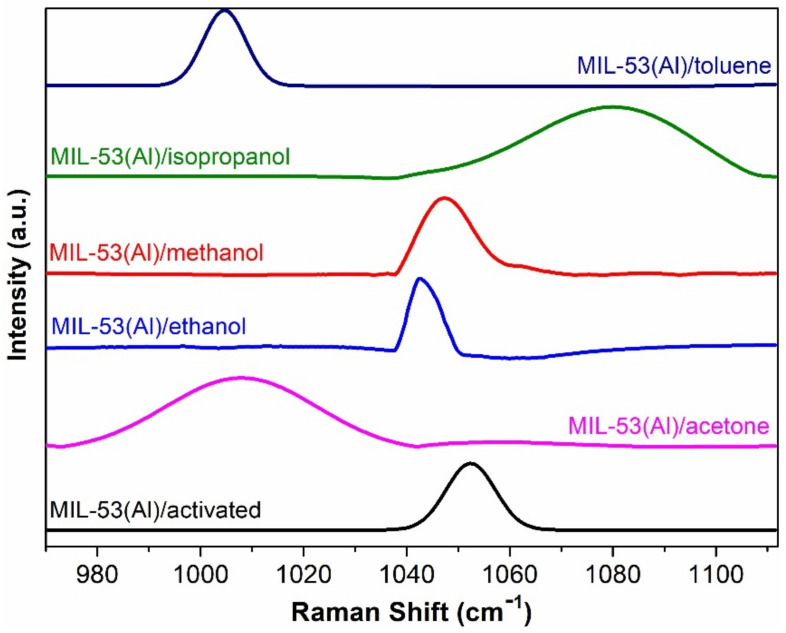
Zoomed view of FT-Raman spectra related to breathing properties of MIL-53(Al)/activated and MIL-53(Al)/VOCs.

The phase transition in MIL-53(Al) is intimately connected with the breathing effect, which is characterized by a shift in the large pore (LP) structure’s vibrational mode from 1026 cm^−1^ to 1017 cm^−1^ in the presence of narrow pore (NP) H_2_O or minor variations in wavenumbers within the closed pore (CP) structure [38,39,40]. Our experimental findings show that this specific wavenumber was observed at 1052 cm^−1^ for MIL-53(Al)/activated and 1034 cm^−1^ for MIL-53(Al)/hydrated, as depicted in Figure 3A. We hypothesize that this shift may be associated with a CH in-plane deformation of the aromatic ring (δCH), dependent on the degree of rotation of the benzene ring, which is often referred to as ring breathing. After adsorption of VOCs, we observe distinct variations in this vibrational mode. In the case of MIL-53(Al)/toluene, the vibrational mode displays a more intense peak within the frequency range of 1004 cm^−1^, whereas, for MIL-53(Al)/acetone, the peak is less intense and peaked at 1010 cm^−1^. Furthermore, for MIL-53(Al)/isopropanol, methanol, and ethanol, this vibrational mode is observed at 1080, 1052, and 1023 cm^−1^, respectively. For better understanding, in Figure 4, we have also displayed the zoomed view of FT-Raman spectra in the range 970–1110 cm^−1^, which is particularly associated with the breathing behavior of MIL-53(Al) under the adsorption of different VOCs. Our analysis also highlights the potential utility of other vibrational modes, which influence the flexibility of the structure, in identifying the aforementioned phase transitions. Particularly, Raman intensities and the shapes of all the spectra vary with respect to the adsorption of each volatile organic compound, respectively.

### 3.4. Time-Resolved Photoluminescence Spectroscopy of MIL-53(Al) MOFs

**Figure 5:**
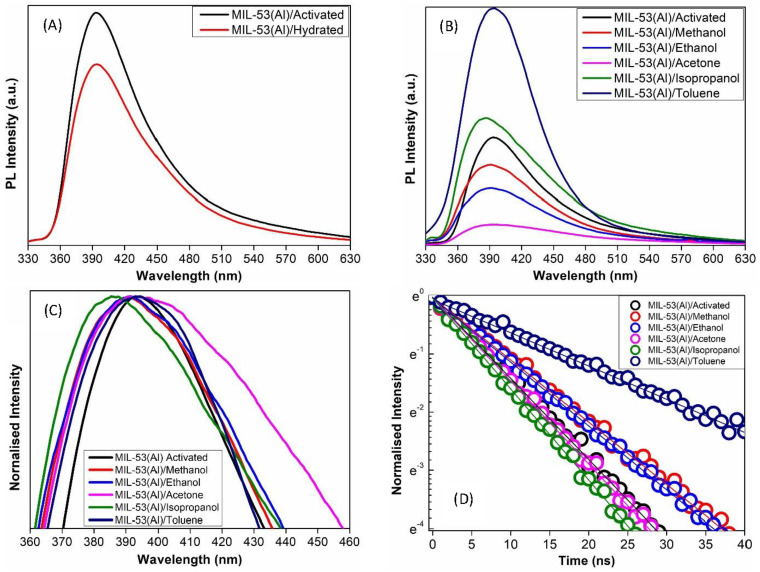
Comparison between photoluminescence (PL) emission spectra of MIL-53(Al)/activated and hydrated (**A**). Emission spectra of MIL-53(Al)/activated and VOCs (**B**). Normalized photoluminescence (PL) spectra of MIL-53(Al)/activated and VOCs to evidence the guest-induced spectral shifts of emission maxima (**C**). Decay curves recorded on the photoluminescence (PL) peaks in MIL-53(Al)/activated and VOCs (**D**). Both emission spectra and decay curves are excited at 305 nm.

Time-resolved photoluminescence (TRPL) spectroscopy spectra of MIL-53(Al) MOFs were performed using an excitation wavelength of 305 nm to provide further insights into the MOF’s response to different adsorbates. Emission spectra reported in Figure 5A–C were acquired by setting T_W_ = 50 ns and T_D_ = 5 ns, while decay curves reported in Figure 5D were acquired by monitoring the photoluminescence (PL) intensity at the emission peak, around 393 nm, setting T_W_ = 1 ns and T_D_ increases from 0 ns in steps of 1 ns until the photoluminescence (PL) was reduced by at least an order of magnitude. The pristine MIL-53(Al)/activated MOF, which serves as the reference, exhibits a peak emission at 393 nm with a lifetime of 7.1 ± 0.3 ns (Figure 5A,D). The presence of water molecules as quenchers reduces the photoluminescence (PL) intensity but does not influence the intrinsic time decay properties, which remain unchanged with a lifetime of 7.1± 0.3 ns (Figure 5A,D). The greatest effects are observed when the MOF is exposed to toluene; the emission intensity increases approximately three times, and its lifetime lengthens to 16.0 ± 0.5 ns (Figure 5B,D). Adsorption of isopropanol in the MOF framework also increases the photoluminescence (PL) intensity, but only slightly, while the lifetime is 6.1 ± 0.3 ns. In contrast, acetone, ethanol, and methanol reduce the photoluminescence (PL) intensity, the more effective quencher is acetone, and the lifetime is 6.5 ± 0.3 ns for MIL-53(Al)/acetone, 9.1 ± 0.4 ns for MIL-53(Al)/methanol, and 8.8 ± 0.4 ns for MIL-53(Al)/ethanol.

As it is clear in Figure 5C, there are also spectral shifts in emission maxima, which signify alterations in the MOF’s electronic structure with the adsorption of VOCs. These findings highlight the sensitivity of MIL-53(Al) MOF’s photon emission to the presence of various VOCs and the significance of the specific interactions between the MOF and adsorbed molecules for the tunability of the MOF’s photoluminescence (PL) properties in sensing and optoelectronic applications.

## 4. Discussion

To address the kinetics of host-guest interactions, it is essential to consider the various factors that may influence the adsorption kinetics of guest molecules within the MOFs: hydrogen bonds, pore size, hydrophobic interactions, cross-sectional area, coordination effects, π-π interactions, polarizability, functional groups, electrostatic interactions, kinetic diameter, and molecular width of the guest molecules [16,41,42,43]. In this study, apart from water adsorption, the VOCs adsorbates are categorized into three groups for a comparative discussion: (i) alcohols (methanol, ethanol, and isopropanol), (ii) aromatics (toluene) and (iii) ketones (acetone), respectively. Regarding water adsorption in MIL-53(Al), the kinetics involve three stages: (i) water molecules forming a hydrogen bond between the oxygen of H_2_O molecule and the hydrogen of µ2-OH groups of the [AlO4(OH)2] octahedra interconnected by terephthalate linkers, (ii) water molecules forming two hydrogen bonds with hydrogen atoms of the H_2_O molecules and an oxygen atom of the terephthalate ligand, and (iii) water molecules with strong guest-guest interactions at the center of the channel. In the large pore (LP) form, adsorbate-adsorbate interactions predominate over adsorbate-adsorbent interactions, while the opposite is true in the narrow pore (NP) form [38,40].

As far as the adsorption of VOCs is concerned, methanol, despite its high polarity, exhibits almost comparable weight loss to that of pristine MIL-53(Al)/activated. Furthermore, the loading quantity of methanol was also found to be less than the rest of the VOCs. This is also consistent with the X-ray analysis of these two samples as well. We reasoned that the interactions between the guest molecules and the MOF framework are primarily responsible for such response. Although methanol can establish some hydrogen bonding interactions, these might typically be weaker than the stronger capabilities of other VOCs, such as acetone, isopropanol, and ethanol. Additionally, methanol is also highly volatile, which may limit its ability to remain inside the MOF cavities for a longer period. As a result, methanol’s interactions with the MIL-53(Al) MOF are less likely to cause significant structural modifications or distortions as well. However, from two weight losses (below 200 °C) associated with ethanol in TGA analysis and from the estimation of the loading quantity of ethanol within the MOF, it is evident that it undergoes two adsorption kinetics with the host framework. Initially, ethanol molecules reside on the surface before diffusing well into the framework’s pores via stronger hydrogen bonding. Isopropanol also interacts with MIL-53(Al) MOF in a similar manner, as the first weight loss (below 100 °C) is associated with the desorption of surface molecules, and the second weight loss (below 200 °C) is attributed to the desorption of strongly bonded isopropanol molecules within the framework. Furthermore, these findings are also consistent with the PXRD analysis, indicating that crystalline structures in both cases remain mostly in the hydrated narrow pore (HyNP) structure with considerable framework contraction. In contrast, acetone, despite its lower polarity, may diffuse well into the MOF cavities due to its smaller kinetic diameter and molecular width, leading to the complete structural transitions into the hydrated narrow pore (HyNP) structure with significant shrinkage of the host framework.

The adsorption kinetics of toluene within the MIL-53(Al) (MOF) possess unique characteristics due to its nonpolar nature and the specific interactions it forms with the MOF structure. Toluene is primarily involved in pi-pi interactions with the MOF, facilitated by its aromatic benzene ring, which is rich in π-electrons. The MOF itself comprises metal centers and aromatic organic linkers with π-electrons, resulting in stronger pi-pi stacking interactions [44,45]. Additionally, van der Waals interactions contribute due to toluene’s nonpolar characteristics, enabling attraction between toluene molecules and the nonpolar regions of the MOF. Although pi-pi interactions play a vital role in stabilizing adsorbed toluene, the observed expansion of the MOF framework in PXRD analysis is more likely due to the MOF’s adaptability to accommodate toluene molecules within its pores. Toluene induces structural adjustments and steric effects on the MOF’s porous network, leading to changes in lattice parameters and peak positions. Our findings are in line with the concept of guest-induced vibrational modes dynamics. As observed using FT-Raman spectroscopy, the adsorption of water molecules and VOCs caused a significant deformation in the benzene ring of the backbone phenyl rotor, which suggests the high flexibility of MIL-53(Al) under the adsorption of different guest molecules.

The guest-induced photoluminescence (PL) properties of MIL-53(Al) MOF were also studied, and differences among the adsorbed VOCs were evidenced. It was observed that, when exposed to water molecules, because of their high polarizability and capacity for hydrogen bonding, water molecules are known to be effective fluorescence quenchers [46]. Water molecules can interact with the excited-state fluorophores in the MOF, resulting in non-radiative decay processes. The emission intensity is decreased owing to the energy transfer mechanism from the excited-state fluorophores to the water molecules. Conversely, the unchanged lifetimes imply that the water content has no appreciable effect on the intrinsic radiative decay mechanism in MIL-53(Al). Henceforth, it indicates that the intrinsic radiative decay process of MIL-53(Al) remains almost constant despite the reduced photoluminescence (PL) intensity caused by water-induced quenching. The exposure to toluene enhances lifetime and photoluminescence (PL) intensity threefold, indicating that toluene molecules interact with the aromatic linker of MIL-53(Al) via pi-pi interactions. Acetone quenches the photoluminescence (PL) due to energy transfer processes, while isopropanol and methanol introduce complex dynamics with altered emission intensities and lifetimes. Ethanol results in a significant PL quenching and spectral shift, indicating changes in the MOF’s electronic structure due to the coordination of the analyte-Al or analyte-oxygen group [24,44,45,46]. It is also worth mentioning that, according to the TRPL analysis, there were two dominant cases. (i) Enhancement in photoluminescence (PL) intensity and lifetime under the adsorption of toluene in MOF and (ii) Photoluminescence (PL) quenching under acetone adsorption in the MOF framework. Most probable reasons for such a response are attributed to the following facts: Toluene’s aromatic ring structure is well defined. Its inflexible, planar shape might enable it to interact or stack with the aromatic rings of the terephthalic acid of the organic linkers. These interactions could modify the MOF’s electronic characteristics in a way that enhances photoluminescence (PL) [47]. Furthermore, resonance may also involve the expanded pi-electron system in toluene, which could improve the photoluminescence PL via electronic effects. On the contrary, acetone has a less planar and more compact structure than toluene. Its flexibility and smaller size may prevent significant π-π interactions with the ligands of the MOF. Alternatively, acetone may introduce electronic states that quench the photoluminescence (PL), or it may occupy the pores without appreciably changing the MOF’s electronic structure. Furthermore, acetone may even provide channels for non-radiative energy transfer that result in photoluminescence (PL) quenching [47]. These findings demonstrate the sensitivity of MIL-53(Al) MOF’s photoluminescence (PL) to different VOCs, offering insights into their interactions and potential applications in sensing and optoelectronics.

## 5. Conclusions

This study provides valuable insights into the host-guest interactions, structural transformations, thermal properties, vibrational dynamics, and photoluminescent responses of MIL-53(Al) MOFs in the presence of various volatile organic compounds (VOCs) and water. The adsorption kinetics and structural changes of MIL-53(Al) are significantly influenced by the molecular size, shape, polarity, and interactions of the guest molecules, as well as the specific characteristics of the MOF framework. The MOF’s distinct response to toluene, marked by pi-pi interactions and structural adaptations, highlights the unique behavior of nonpolar VOCs within the MOF structure. The intricate guest-induced photoluminescence (PL) dynamics further demonstrate the sensitivity of MIL-53(Al) MOF to different VOCs, particularly turn-on and turn-off photoluminescence (PL) mechanisms in the case of toluene and acetone adsorption are offering potential for applications in sensing and optoelectronics. This study contributes to the fundamental understanding of MOF-guest interactions and their relevance for real-time sensing applications, and it underscores the importance of tailoring MOF properties for specific applications by choosing suitable guest molecules. The findings presented here expand our knowledge of MIL-53(Al) MOF response and open doors to a range of innovative applications in areas such as optical sensing, catalysis, and separation processes.

## Data Availability

Upon reasonable request, the corresponding author will provide the data supporting the study’s findings.
